# Finite Element Analysis Investigate Pulmonary Autograft Root and Leaflet Stresses to Understand Late Durability of Ross Operation

**DOI:** 10.3390/biomimetics5030037

**Published:** 2020-08-03

**Authors:** Francesco Nappi, Antonio Nenna, Francesca Lemmo, Massimo Chello, Juan Carlos Chachques, Christophe Acar, Domenico Larobina

**Affiliations:** 1 Department of Cardiac Surgery, Centre Cardiologique du Nord de Saint-Denis, 93200 Paris, France; 2 Department of Cardiovascular Surgery, University Campus Bio-Medico of Rome, 00128 Rome, Italy; antonio.nnn@hotmail.it (A.N.); M.Chello@unicampus.it (M.C.); 3 Faculty of Engineering, University of Turin, 10124 Turin, Italy; chiccalemmo1997@gmail.com; 4 Department of Cardiovascular Surgery Carpentier Foundation, Pompidou Hospital, University Paris Descartes, 75015 Paris, France; j.chachques@aphp.fr; 5 Department of Cardiovascular Surgery, Hopital de la Salpetriere, 75013 Paris, France; c.acar@psl.aphp.fr; 6 Institute for Polymers, Composites, and Biomaterials, National Research Council of Italy, 00185 Rome, Italy; larobina@unina.it

**Keywords:** pulmonary autograft, bioinspired Ross operation, pulmonary autograft expansion, pulmonary autograft biomechanical

## Abstract

Ross operation might be a valid option for congenital and acquired left ventricular outflow tract disease in selected cases. As the pulmonary autograft is a living substitute for the aortic root that bioinspired the Ross operation, we have created an experimental animal model in which the vital capacity of the pulmonary autograft (PA) has been studied during physiological growth. The present study aims to determine any increased stresses in PA root and leaflet compared to the similar components of the native aorta. An animal model and a mathematical analysis using finite element analysis have been used for the purpose of this manuscript. The results of this study advance our understanding of the relative benefits of pulmonary autograft for the management of severe aortic valve disease. However, it launches a warning about the importance of the choice of the length of the conduits as mechanical deformation, and, therefore, potential failure, increases with the length of the segment subjected to stress. Understanding PA root and leaflet stresses is the first step toward understanding PA durability and the regions prone to dilatation, ultimately to refine the best implant technique.

## 1. Introduction

International guidelines and position papers from professional societies recommend Ross operation as a valid option for congenital and acquired left ventricular outflow tract disease in selected cases [1,2,3,4,5,6,7,8]. Patients who benefit most from this procedure are children and young adults, women of childbearing age, and patients with contraindications to oral anticoagulants [9,10,11,12,13]. The advantages are related to the somatic growth of the cardiovascular structures and with the avoidance of anticoagulants that would be required lifelong in the case of conventional mechanical prostheses [14,15,16,17,18]. However, the incidence of pulmonary autograft (PA) expansion reported after Ross operation, without loss of integrity of the valve leaflets, varies from 20% to 40%, and reoperation is not uncommon [19,20,21,22,23,24,25,26,27,28,29,30].

As the pulmonary autograft is a living substitute for the aortic root that bioinspired the Ross operation, we have created an experimental animal model in which the vital capacity of the PA has been studied during physiological growth. Therefore, we have reinforced the PA with resorbable scaffolds or semi-absorbable composite prostheses capable of mediating a biomechanical effect and counteracting the abnormal process of the extracellular matrix disruption leading to PA dilatation when the conduit is subjected to systemic pressure. We further revealed the mechanisms of growth, remodeling, and stress shielding of the reinforced PA by means of an experimental large animal model supported by an ex vivo mathematical and physical model [31,32,33,34,35,36,37,38].

This study aims to integrate the animal pattern with mathematical models from biomechanics. In detail, the present study aims to determine any increased stresses in PA root and leaflet compared to the similar components of the native aorta. Understanding PA root and leaflet stresses is the first step toward understanding PA durability and the regions prone to dilatation, ultimately to refine the best implant technique. First, we assumed the nonlinear constitutive stress–strain relationship, as evidenced by the mechanical tests, to examine the mechanical differences between the two vessels along the circumferential and the longitudinal directions [35,39]. Second, a hexahedral regular mesh was generated, each finite element being associated with eight nodes with three translational degrees of freedom, to measure the expansion of PA [38]. Third, 3D ideal reinforced pulmonary autograft with a composite semi-resorbable device was designed to prevent degeneration and failure of PA [32,33,34,37]. Finally, above all, we tried to explain the relationship between the pathological process that occurs in the PA wall and the stress levels to which the pulmonary autograft is exposed. We thus explained the mechanisms underlying the structural integrity and flexibility of the PA, with particular regard to the balance between apoptosis and cell proliferation of vascular smooth muscle cells in conditions of high stress levels [36]. The final results of the regulatory remodeling pathways of the extracellular matrix within the PA reinforced with a semi-absorbable scaffold are described in the presence of high stress–strain condition both in valve leaflet and root [36].

### The “Ross Experimental Project”

We developed a “Ross experimental project” that is a European partnership of investigators who aim to provide the basis for studying how to prevent the expansion of pulmonary autograft used in aortic valve surgery. The project was initiated in January 2011 and required the collaboration of the Department of Cardiac Surgery of Centre Cardiologique du Nord, la Pitie Salpetriere Hospital and the Institute of Cardiovascular and Medical Sciences, University of Glasgow.

The primary objective of the Ross experimental project was to combine the individual data of the experimental animal model by comparing nonreinforced and reinforced pulmonary autograft to provide an ideal substitute for aortic valve surgery. Using an experimental model of growing sheep based on the simulation of the Ross operation, the experimental project Ross estimated that the analysis of the results would have detected significant differences in the pulmonary autograft morpho-structure at the 6-month follow-up. The pulmonary autograft was inserted in the descending aorta, while the right ventricle outflow tract was reconstructed with a fresh homograft from another lamb of the same age and weight or native pericardial neo-conduit [32,33,34,37].

In the first experimental studies, the comparison between reinforced and nonreinforced pulmonary autograft took into account the variable of somatic growth that is fundamental when the Ross operation is performed during the patient’s growing age [35,36,39]. In the human series, although the rate of superior expansion of nonreinforced pulmonary transplant has been established, there is a paucity of data that report a potential clinical benefit for late outcomes over 20 years when comparing reinforced vs. nonreinforced Ross procedure [5,21,22,24,25,29,30].

The results were presented and discussed in June 2013 at the Seventh Biennial Congress of the Society for Heart Valve Disease and Heart Valve Society of America in Venice (Italy) [27]. Subsequently, the prototype of a semi-absorbable scaffold was discussed in March 2018 at the American College of Cardiology [8,31], and, immediately afterward, the European patent was granted (15791770.9-1109/3215196).

## 2. Methods

### 2.1. Experimental Animal Model

#### 2.1.1. Implantation

**Figure 1:**
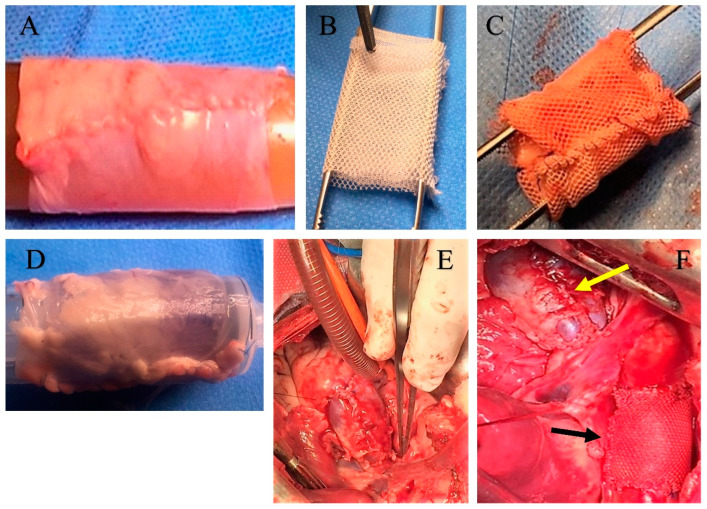
Pulmonary autograft before and after implantation. (**A**) Pulmonary autograft before implantation; (**B**) External expanded polytetrafluoroethylene (e-PTFE) reinforcement. (**C**) Pulmonary autograft reinforced with external e-PTFE. (**D**) Pericardial patch used for reconstruction of right ventricular outflow tract (RVOT); (**E**) Neopulmonary trunk implanted; (**F**) Pulmonary autograft reinforced with e-PTFE (black arrow) and neopulmonary trunk (yellow arrow) implanted.

**Figure 2:**
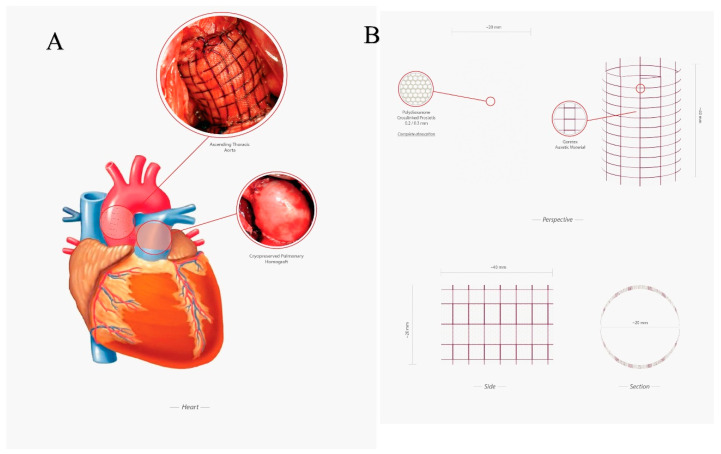
(**A**) Experimental model of Ross operation. Ascending aorta was replaced using a pulmonary autograft reinforced with semiresorbable prosthesis (up), the right outflow tract was reconstructed with the use of a cryopreserved pulmonary homograft (down); (**B**) Semiresorbable crosslinked prosthesis made by an external layer of e-PTFE and an internal layer of polydioxanone.

The technical details of the experimental model of Ross Operation have been previously described [33,34,37]. Briefly, all lambs were treated according to the European regulations for animal experimentation. After premedication with ketamine (25 mg/kg IM), anesthesia was induced with 10 mg/kg of thiopental sodium via the internal jugular vein and was maintained with halothane or isoflurane (1% to 2%, 5%) in mechanically ventilated lambs. An intravenous bolus of 100 mg of lidocaine was used as prophylaxis against rhythm disturbance. The electrocardiogram was monitored. The chest was prepped and shaved. The heart was approached via left thoracotomy, and the trunk of the pulmonary artery was dissected free from its right ventricular origin up to its bifurcation in the pulmonary arteries. Approximately 9 cm of the descending thoracic aorta was left for clamp positioning and to easily perform the anastomosis with the pulmonary artery trunk. A cardiopulmonary bypass was established between the right atrium and descending thoracic aorta after injection of 3 mg/kg of heparin given intravenously. The cerebral circulation of the animal was guaranteed on a beating heart. Four centimeters of the pulmonary artery trunk was transposed into the descending aorta with an end-to-end anastomosis in 5-0 prolene. The right outflow tract was reconstructed by insertion of a fresh pulmonary artery homograft explanted from animals sacrificed on the same day for another experimental study. The left thoracotomy was closed, and aspiration drainage left in place. All experiments were performed in respect of guidelines for animal care and handling. The protocol was approved by the institutional animal care committee (Figure 1A–F and Figure 2).

#### 2.1.2. Preparation of the Reinforcing Material

A group of *n* = 10 lambs received the prosthetic reinforcement of the PA. The mesh was cut into a rectangle measuring 20 mm in height corresponding to the height of the pulmonary autograft, rolled on a metallic candle, and then fixed with a suture, so as to create a cylinder with an internal diameter of 10 mm (20 mm height and 10 mm diameter, as illustrated in Figure 1A–D). The autograft was then inserted into the fibrillar cylinder and was anastomosed, suturing both its margins and those of prosthetic structure to the PA trunk. A number of 10 animals did not receive any reinforcement and served as control nonreinforced group. All animal experiments were performed with respect to guidelines for animal care and handling, and the protocol was approved by the institutional animal care committee Figure 1A–D and Figure 2A,B.

#### 2.1.3. Specimen Collection and Characteristics

Thirty-six mechanical stress–stretch tests were performed on 3.5 cm of animal explanted pulmonary (*n* = 6) and aortic root (*n* = 6) preserving the native valve. All samples were retrieved from the heart under standard aseptic conditions in a multi-organ heart beating donors’ (MOHBD) study cohort. All hearts were treated at the Saint Louis Tissue Bank, and processing started within 24 h after procurement. Tissue preparation was carried out in a clean room of Class A with vertical laminar flow in a Class C environment following the quality standards for tissues and cells strictly. After dissection, the heart valves and conduit were macroscopically examined, and those that were considered suitable for implant were immersed in a solution of an antibiotic cocktail. Three different types of antibiotics were added to 500 mL of RPMI 1680 (Invitrogen, France) to a final concentration of 320 mg/L gentamicine (Panpharma, Fougères, France), 500 mg/L vancomycine (Merck Génériques, Lyon, France), and 600 mg/L clindamycine (Pfizer, Paris, France). The antibiotic incubation proceeded for a minimum of 20 h at 4 °C. After decontamination, tissues were measured, transferred into a cryoprotective solution of 10% DMSO (WAK, Steinbach, Germany) and 20% human albumin (Albunorm 5%, Octapharma, Boulogne Billancourt, France) in RPMI, and sealed in a double pouch (NPBI/Fresenius, France). The pouches were frozen in a program-controlled-rate freezer until −160 °C and then stored at the vapor phase of liquid nitrogen in a storage tank (−150 to −180 °C).

In addition to the other measures for safety, such as donor file review and serology safety, samples for bacteriological culture were routinely collected from all the tissues at two different steps: (1) at the beginning of tissue processing (samples from myocardium and transport solution); (2) after antibiotic incubation (samples from myocardium). Samples (1) and (2) were added to a Schaëdler broth (Biomerieux, Craponne, France) and incubated for 10 days at 37 °C. The liquid medium was then homogenized and seeded; on the one hand into Chocolate agar with PolyViteX (Biomérieux SA, Craponne, France) for aerobic culture, Columbia agar + 5% sheep blood (Biomérieux SA, Craponne, France) for anaerobic culture, and Sabouraud Chloramphenicol Gentamicin agar (Bio-Rad Inc., Hercules, CA, USA) for fungal isolation and on the other hand, in aerobic and anaerobic BactA/ALERT culture media (Biomerieux, Craponne, France). The tissues with positive microbiological results in the post-antibiotic treatment were rejected. Frozen heart valves were thawed in a 40°C thermostatic bath and rinsed several times with a DMSO gradient in saline.

### 2.2. Constitutive Model and Material Properties

The samples were tested to evaluate the mechanical properties in tension mode by a Dynamic Mechanical Apparatus (Q-800 TA instruments, New Castle, DE, USA), which guarantees precise control of stress, and low friction support. In detail, a total of 36 specimens were cut from each of the 12 human explanted roots. Among the 36 samples, 12 of 4 mm in width and 8 mm in length were collected from the valve leaflet, while 24 approximately 10 mm in width and 12 mm in length were taken from the wall of the roots. The latter was further divided as follows: 12 were obtained by cutting the roots longitudinally with respect to the root axis, while 12 were cut circumferentially. All lengths and widths were measured by a caliper (Mitutoyo model. 500–457) with the specimens mounted on the apparatus, while thicknesses were evaluated by a digital micrometer (Mitutoyo IP65) before mounting the sample. The nonlinear behavior of all samples was assessed in a force-controlled test up to about 240 kPa for the leaflet and 120 kPa for the walls. Appropriate grip insured constant contact with the sample, avoiding any undesired slip effect. The results are reported in terms of average engineering stress defined as force with respect to the initial cross-sectional area vs. average stretch ratio, including as actual over initial sample height.

The measured forces (*f*) were converted to stresses (*σ*) in the principal directions (circumferential θ and longitudinal *L*) by Equations (1) and (2):

(1) $$\sigma_{\theta \theta }=\lambda_{\theta }\frac{f_{\theta }}{t\;l_{L}}$$

(2) $$\sigma_{LL}=\lambda_{L}\frac{f_{L}}{t\;l_{\theta }}$$

where *t* is the thickness and *λ* represents the stretch ratio (Equation (3)):

(3) $$\lambda =\frac{l\left(t\right)}{l\left(0\right)}$$

In the equation above, l(t) is the deformed length, while l(0) is the resting tissue.

The material’s response to stress was described mathematically using an Ogden-type hyperelastic material. In the Ogden model, the strain energy density is expressed in terms of the principal stretches $\lambda_{i}\;i=1,2,3$ as Equation (4):

(4) $$W\left(\lambda_{1},\lambda_{2},\lambda_{3}\right)=\overset{3}{\underset{i=1}{\sum }}\frac{\mu_{i}}{\alpha_{i}}\left(\lambda_{1}^{\alpha_{i}}+\lambda_{2}^{\alpha_{i}}+\lambda_{3}^{\alpha_{i}}-3\right)$$

where $\mu_{i}$ $\alpha_{i}$ are material parameters. Stress–stretch relations can then be computed by simply deriving *W* with respect to $\lambda_{i}$; for an incompressible material under uniaxial tension, the relation reads Equation (5):

(5) $$\sigma =\overset{3}{\underset{i=1}{\sum }}\mu_{i}\left(\lambda^{\alpha_{i}}-\lambda^{-\frac{1}{2}\alpha_{i}}\right)$$

The specific values of the parameters $\mu_{i}$ and $\alpha_{i}$ being reported in the table at the bottom of Figure 5C, for the cases at hand.

#### 2.2.1. FE Simulations

**Figure 3:**
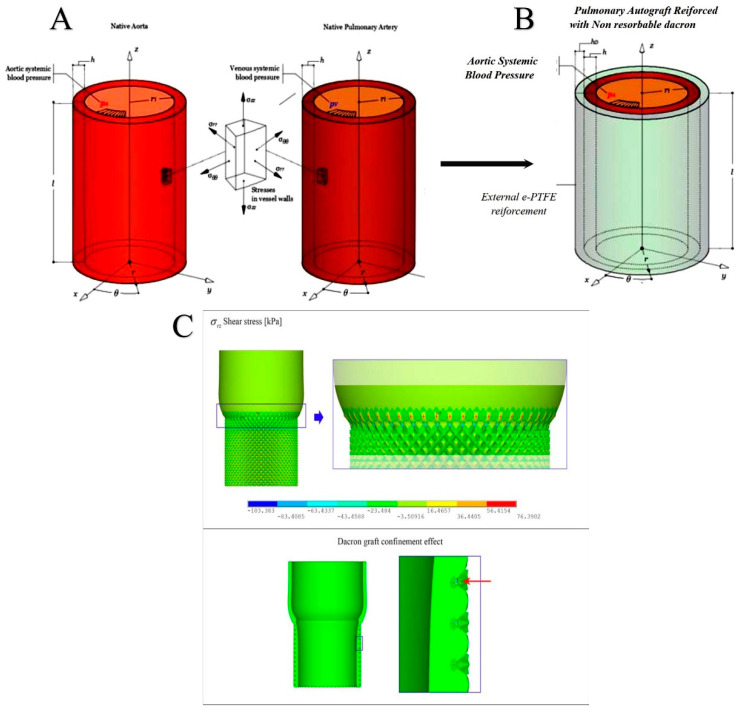
Geometry and the mechanical features of the native aorta and the native pulmonary artery (**A**) and the pulmonary autograft (**B**). (**C**) Illustration of the spurious shear stresses in the suture zone (Top), and the Dacron structure confinement acting on the pulmonary autograft (PA) pressurized vessel (Bottom).

**Figure 4:**
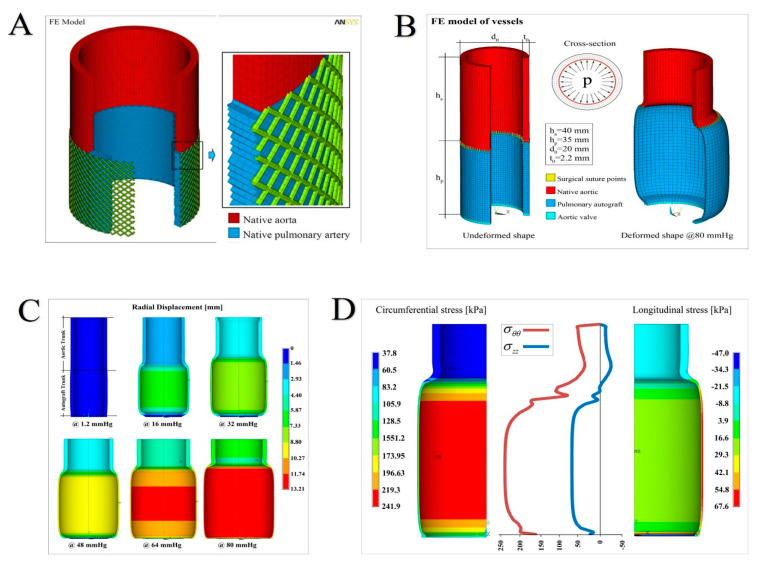
(**A**) Pulmonary autograft reinforced with semiresorbable prosthetics integrated into the pulmonary autograft wall. Right. The geometry of the finite element (FE) model, with a detail showing the communication of the native aorta (in red) with the pulmonary artery tract (blue), integrated with the external e-PTFE structure at the time of full development (BSA 2.2) (**B**) Overall sketch of the finite element model reconstruction of the aorta–suture–autograft–annulus ensemble: undeformed system (left); deformed (at the maximum pressure level) model (right) and cross-section with applied pressure. At the bottom, the legend with the details of the elements used and distinguished for material properties. (**C**) Sequence of deformations at increasing pressure levels up to 80 mmHg. The contour plots refer to the displacements along the radial direction (in mm). (**D**) Hoop (circumferential) and longitudinal (axial) stress profiles as a function of the vessel axis (middle), with contour plot details showing the spatially inhomogeneous distribution of the stresses (in kPa).

The Ogden constitutive equation was first uploaded into the finite element (FE) library of the code ANSYS (ANSYS 13.0), the utilized advanced commercial FEM-based code (Ansys 13.0 user’s manual, 2009), where a cylindrical geometry was utilized to describe both aorta and PA roots. Thickness, height, and diameter of both aorta and PA conduits were taken from the average undeformed states directly measured on the samples. To ensure accurate results, a hexahedral regular mesh was generated, each finite element being associated with eight nodes with three translational degrees of freedom. In particular, an ad-hoc custom made Ansys procedure, written in APDL parametric language, was developed to reconstruct the FE model of the whole system constituted by the aorta tract connected with the pulmonary autograft by means of the suture region. Figure 3 and Figure 4A–C illustrate the 3D reconstruction of the geometrical model, with the mesh obtained by means of about 18,000 standard hexahedral (SOLID185) elements and almost 25,000 nodes with three degrees of freedom for each node. The suture was also built up in detail by means of 1D elastic link elements (LINK180). The dynamic interaction between leaflets and blood flow being the scope behind of the present work, we decided to model the influence of leaflets and annulus on the mechanics of the autograft by simply substituting them with an equivalent virtual ring at the basis of the model with material properties taken from the experimental results of the valve leaflets (see cyan region in Figure 4B). The model was thus loaded with a typical mean pressure value of 80 mmHg (about 10.61 kPa) applied on the internal surface of the blood vessels and—to replicate a faithful situation—suitable symmetrical boundary conditions were considered on both top and bottom cross-sections of the structure. The FE analyses were then performed in static nonlinear regimes, both in terms of constitutive laws (hyperelasticity) and geometry (large deformations), employing standard convergence algorithms.

#### 2.2.2. Comparative Physical-Mathematical Model

**Figure 5:**
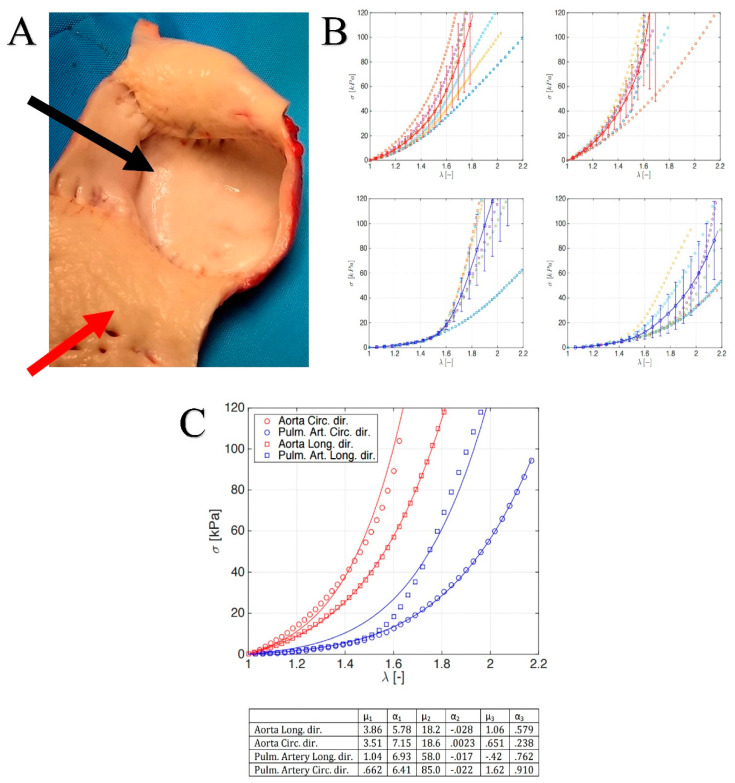
(**A**) Pulmonary autograft (black arrow) and aorta explanted (red arrow) at 1 year. (**B**) Stress–stretch curves for aorta: longitudinal (top-left) and circumferential (top-right) direction; stress–stretch curves for pulmonary artery: longitudinal (bottom-left) and circumferential (bottom-right) direction. (**C**) Synoptic of the (top) average stress–stretch curves for both aorta and pulmonary artery along the two mechanically relevant directions (see legend for details) and (bottom) Table with the fitting parameters.

We developed an ad-hoc mathematical model to simulate the biomechanical response in terms of growth and remodeling of pulmonary arteries under systemic pressure during physiological growth, in both reinforced and nonreinforced conditions [33,34,37]. The concept of an innovative prosthesis system realized by combining bioresorbable and auxetic synthetic materials is supported by the above-mentioned mathematical model. This pattern describes the evolutive behaviors of both the internal structure of the vessel walls (intima, media, and adventitia) and the PA composite reinforcement constituted by a biodegradable scaffold made of polydioxanone (PDS), integrated with an external GORE-TEX weave (expanded polytetrafluoroethylene, e-PTFE) whose structure exhibits auxetic (i.e., negative Poisson’s ratio) behavior (see Figure 2 and Figure 5). The evidence demonstrated in the biomechanical model and supporting the synergic effects of the two synthetic materials includes the following observations: the mechanisms of stress shielding simultaneously ensure the autograft integrity, its progressive and controlled dilation, allowing regional somatic growth and preventing aneurismal degeneration [35,36,38,39].

Physical evaluation revealed mechanical stress associated with progressive overstrain in the pulmonary artery wall under systemic pressure that might affect PA integrity and the endothelialization process [34,36,39]. The prevention of the graft dilation is, therefore, crucial, and evidence based on the analysis of the mathematical model attributes the role of director of the process to the phenomenon of stress-shielding. The double reinforcement of pulmonary autograft avoids the mechanical overload of the PA walls preventing an excessive deformation prodromal to aneurysmal dilation. The histologic changes observed were the arterialization process and the thickening of the PA.

The degradation process of the PDS-scaffold gradually reduced its mechanical contribution. The feedback was translated from the wall of the PA as a signal to start a stress-compatible progressive remodeling of the vessel. The result led to physiological changes in tissue microstructure and to achieve the mechanical properties required to support systemic pressures. Once the bioresorbable scaffold had completed its degradation program and the strengthened vessel walls could actively respond to the systolic pressure, the expanded Polytetrafluorethylen (e-PTFE) structure accompanied PA media and adventitia toward their progressive aortic somatic growth, by stretching its weave to gain stiffness and to confine further vessel expansion effectively, so avoiding tissue prolapse and aneurismal degenerative phenomena.

To mathematically describe the above-observed processes, three biomechanical modeling cases can be recognized:

- Reference Aorta, regarding the modeling of an aortic tract subjected to internal systemic pressure, say pi, of 120 mmHg (16 kPa, assumed constant). This case establishes for the reference aorta selected benchmark quantities, say the physiological growth over a six-month period (represented by the evolution of both diameter and thickness of the vessel layers) and the wall mechanical stresses (Figure 3A).
- No Reinforcement, analyzing the case of a not reinforced pulmonary artery transposed into aortic position at the pressure pi and subjected to growth and remodeling processes. The results of this simulation are directly compared with the outcome of the control group of the animal model (Figure 3B).
- Composite Reinforcement, concerning the mechanical analysis of the reinforced PA system undergoing growth and remodeling. The presence of the prosthesis is simulated by integrating the mechanical properties of the adventitia with those of the PDS biodegradable structure, by thus additionally providing an external variable pressure *po*, accounting for the e-PTFE armor elastic confinement whose value depends on the armor constitutive properties and evolves as a function of the pulmonary artery dilatation and growth (Figure 2, Figure 5A,B).

## 3. Results

### 3.1. Reinforcement of Pulmonary Autograft

To note that, at 6 months, animal weight was doubled (27 ± 5 kg at day 0, and 55 ± 10 kg at 6 months), suggesting a normal growth process. There were no significant differences in expansion of the PA between no reinforced vs. reinforced Ross with external nonresorbable polyester (20 ± 1 mm vs. 19 ± 2 mm; index ratio, 1.05; *p* = 0.4) but with the almost exponential increase in the expansion of pulmonary autograft (42%) in reinforced Ross with bioresorbable vascular scaffold (BVS/Polydioxanone) and with semi bioresorbable vascular scaffold, which combines the Polydioxanone (PDS) and e-PTFE (28 ± 2 mm vs. 19 ± 2 and 27 ± 2 mm vs. 19 ± 2; index ratio 1.42, respectively). In the reinforced Ross with a semi bioresorbable vascular scaffold, the PA behaved similar to the normal aorta matching the somatic growth. This was the first time in the history of the Ross operation that stress shielding, growth, and remodeling of the pulmonary autograft were studied by a mathematical and biomechanical analysis leading to a better understanding of the biological potential of pulmonary autograft.

### 3.2. Histology of Remodeling of Pulmonary Autograft

Micro- and macroscopic results showed that the interaction between temporary bioresorbable reinforcement and pulmonary autograft orchestrated a complex vascular remodeling process based on a balance between inflammation and production of the extracellular matrix, resulting after biomaterial resorption, in a “neovessel”, which has characteristics similar to those of the aorta but still biologically alive and capable of growing. The use of resorbable polyester was also associated with higher production of the new extracellular matrix that was mainly characterized by a higher content of elastin fiber in the PA, as well as by a more compact organization of collagen fibers in the elastic zone of the vessel. Interestingly, the metalloprotease MMP-9 was found to be overexpressed, indicating an ongoing matrix remodeling process. In parallel, cell proliferation was found to be increased in this group as testified by the significantly higher percentage of ki67 positive cells (26.89% +/− 8.4% in the nonreinforced vs. 51.55% +/− 9.7% in the reinforced group *p* < 0.05). These findings were coupled with a significant reduction in apoptosis in the reinforced Ross, supporting the idea of an active remodeling process in this group (47.8% +/− 7.2% in the nonreinforced Ross vs. 17.5% +/− 5.1% in the reinforced group, *p* < 0.05).

### 3.3. Leaflet and Root Stress

**Figure 6:**
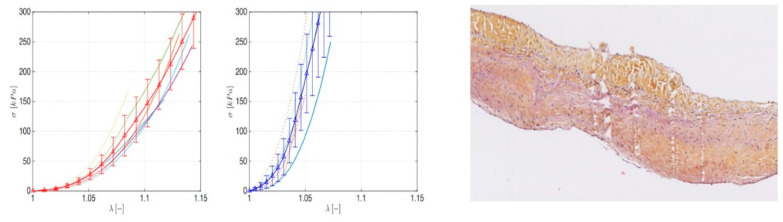
**Left** panel: Stress–stretch curves for pulmonary (left) and aorta (right) leaflet. **Right** panel: Human pulmonary valve explanted 22 years after Ross operation shows a small sector of fibrous densification.

In Figure 5B, we report the uni-axial tests along the mechanically relevant directions, i.e., the longitudinal and circumferential ones, measured for the aorta and PA roots. From the stress–strain profiles, it emerged that the hyperelastic responses of both the vessels were anisotropic, with a classical increasing slope as the stretch grows. By comparing the aorta and PA, it is possible to highlight that the former exhibited a stiffer behavior in both the hoop and axial directions, in line with the consolidated literature. Additionally, strength values seemed to confirm the mechanical resistance hierarchy, being a higher stress threshold in the aorta with respect to PA counterpart. A biomechanically relevant result concerned the stress–strain response of the aorta and PA valve leaflets. In fact, as highlighted in Figure 6 Left panel, very similar qualitative and quantitative behaviors were exhibited by both tissues up to applied forces and prescribed stretches. This would explain the mechanical resistance and durability of pulmonary artery valves when transposed in aorta position in Ross operation, where PA is solicited by high-pressure regimes.

To gain further insights into the actual biomechanical response of native aorta and PA conduits when subjected to physiological pressures, nonlinear finite element (FE) analyses were performed as above described, by ad-hoc building up a parametric model of the vessel tracts. By preliminarily interpolating the experimental stress–stretch curves, we, therefore, uploaded the hyperelastic behavior in the FE model, the results of the fitting being reported in detail in Figure 5C where both the curves and the tables with the adopted parameters are illustrated.

Increasing pressure values within the physiological range were considered for the analyses up to 80 mmHg, corresponding to a (circumferential) maximum stress in PA of about 240 kPa and a diameter dilation overcoming two times the undeformed one (Figure 4B), coherently with the experimental stress–strain measures. The simulations outcomes are synoptically reported in Figure 4C,D.

In particular, Figure 4C collects the sequence of overall deformation of the system with increasing applied pressure. The contour plots show how the radial displacements nonlinearly grew with the exerted pressures, generating significant strain gradients along the longitudinal direction (i.e., the vessel axis) that can be traced as the main responsible for aneurismatic deformations. It has to be noticed that the bulging shape of the deformed autograft, induced by both the discrepancy in stiffness between aorta and PA and the constraint of the basal annulus, determined radial displacement gradients associated with a migration of the suture section upwards, as a result of the competition with the adjacent aorta. In addition, we highlight that the analyses conducted at a pressure higher than about 80 mmHg would kindle extremely localized strains and instability phenomena in correspondence of the suture regions, a fact that confirms the expected inelastic (irreversible) deformation processes prodromal to tissue damaging and failure in the absence of any PA reinforcement. This is emphasized in Figure 4D, where the corresponding hoop stress distribution, at the maximum pressure level, exhibited a strong variation along the vessel axis (z-direction), with localized stress gradients at the PA-aorta connection where the suture was present (Figure 3C). Additionally, as a consequence of the prescribed boundary conditions, the longitudinal stresses displayed a change in sign along the vessel axis, passing from tensile regimes in PA to low compressive values in the aorta tract.

Overall, the results of the FE analyses showed that the realistic response of the aorta-autograft ensemble cannot be captured by means of modeling simplifications, such as numerical simulations performed by taking into account the two vessels separately. As a matter of fact, the FE outcomes demonstrated that the interplay among material properties of the autograft and aorta, suture regions, geometry, and dilation constraints imposed by the annulus is crucial for determining the effects that actual stress concentrations, strain localization onsets, and deformation gradients have on the success of the Ross operation.

## 4. Discussion

In this study, the biomechanical properties of leaflets and arterial root were evaluated, with particular regard to monoaxial circumferential and longitudinal stress to which pulmonary autograft is subjected after its transposition in aortic position with increased pressure stress.

The choice to perform a monoaxial stress test was based on a geometrical tetrahedron analysis. Considering a tetrahedron isolated around a point x, the ideal would be the analysis of the three planes parallel to the coordinated planes passing through the point considered. Otherwise, there is no difference between single-axis analysis and biaxial analysis.

Therefore, we compared the results for both leaflet and conducts of PA and aorta to evaluate the mechanisms of PA failure daunting the long durability of Ross operation. We have generated an ideal model of Ross intervention in which the autograft was reinforced by material that did not affect growth and functionality.

Our finding is in agreement with the previous study of Carr-White et al. [40] about the mechanical behavior of PA after the monoaxial stress test and deepens the previous report of Horer et al. [14], marking a definitive point concerning the nonlinear behavior between growth, remodeling, and shielding stress of pulmonary autograft transposed in systemic regimen pressure. Moreover, our results are in agreement with Skillington et al. [25] that support the use of the inclusion technique to prevent pulmonary autograft expansion.

PA failure and valve regurgitation can lead to severe left ventricular dysfunction, which is a delicate problem that impairs outcomes and quality of life after Ross operation. Pulmonary autograft valve regurgitation has been reported to have an incidence of 40% after 20 years from procedure [19,20] with a freedom from pulmonary valve dysfunction of 53.5% [6,30]. The preferred treatment strategy to prevent dilatation has still not been established. Some observational studies support the benefits of adding an external reinforcement to PA [13,15,38], while other authors report neutral findings [41]. The importance of the biomechanics features of PA in this context appears crucial. One report from Horer et al. stated that there is not homogeneous dilatation of annulus, Valsalva sinus, and sinotubular junction, and an external barrier to protect these zones could be beneficial to avoid expansion of the pulmonary conduit [14]. Mookhoek et al. [42,43] confirmed the nonlinear response to mechanical load typical of the biomechanical characteristics of healthy human arterial tissue in failed pulmonary autografts but warned about increased compliance and reduced wall stiffness of the PA in comparison to native pulmonary artery, providing an explanation for PA dilation [43]. In another study by the same group comparing failed PA root to native aorta, these authors confirmed the increased compliance of explanted PA roots in comparison to the aorta, but, notwithstanding the similar wall thickness, demonstrated the biomechanical remodeling inadequacy of the PA, which was characterized by a significant decrease in wall stiffness in comparison to native aorta [42].

We have already investigated the importance of the remodeling phenomena occurring in the PA and the reflexes on the biomechanical properties of the remodeled autograft by means an in vivo model of Ross operation and also reported the stress shielding effects that a resorbable mesh might exert when applied as a reinforcement for the PA [32,33,34,35,37]. In another report, we previously demonstrated Dacron graft and other synthetic polyesters severely impair aortic compliance when used as external pulmonary artery reinforcement and elicit a strong inflammatory reaction with significant damage to the vessel wall and negative stress shielding effect on distal suture [39]. Moreover, we have highlighted a characteristic morphostructural modification in pulmonary autograft reinforced with polydioxanone. The major histological findings were: vascular remodeling, overexpression of MMP-9, and significantly increased the higher percentage of ki67 positive cells [36]. The remodeling process was shown by the resorbable layer integrated with the PA wall and induced the deposition of new extracellular matrix mainly characterized by elastic fibers. Similarly, we found increased content of elastin fiber at Mallory’s staining in the reinforced group with respect to control as well as a more compact organization of collagen fibers in the elastic zone of the vessel. Moreover, MMP-9 was found to be overexpressed in the reinforced group, indicating an ongoing matrix remodeling process. In parallel, cell proliferation was found to be increased in this group, as testified by the significantly higher percentage of ki67 positive cells. These findings were coupled with a significant reduction in apoptosis in the reinforced PA, supporting the idea of an active remodeling process [37,44,45,46], suggesting a marked difference to the use of Dacron graft [47].

### 4.1. Biomechanics and Finite Element Analysis of Pulmonary Autograft Leaflet and Root

Results of the FE analyses showed that the realistic response of the aorta-autograft ensemble cannot be captured by means of modeling simplifications, such as numerical simulations performed by taking into account the two vessels as separate entities [42,43,44]. The FE outcomes demonstrate that the interplay among material properties of the autograft and aorta, suture regions, geometry, and dilation constraints imposed by the annulus is crucial for determining the effects that actual stress concentrations, strain localization onsets, and deformation gradients have on the success of the Ross operation [38,39].

We added to the equation other important parameters regarding the difference in circumferential and longitudinal stress-bearing abilities of the aorta and the PA and the topographical differences of the biomechanical properties among the components of the aortic root. Indeed, while the aorta revealed a consensual increase in stress and deformation in both the directions, the pulmonary showed better adaptability in the longitudinal direction and a steeper curve in the circumferential response, suggesting the ability of the PA to evenly absorb mechanical stresses and potentially explaining the known dilatation tendency of the pulmonary over time. Second, a higher degree of resistance to deformation of the valve leaflets with a stiffer behavior with respect to the aorta for applied loads of about 240 kPa (1800 mmHg) was demonstrated. The significant physiological pressure value considered for the analyses was 80 mmHg, as also reported by Mookhoek et al. [42,43], and the FE analyses were conducted in a static regime, so neglecting inertia effects, and standard convergence algorithms were also used to follow the nonlinear procedure related to the presence of both large deformations and hyperelastic behavior. The simulations outcomes were synoptically reported, in terms of average hoop stresses and final deformed thickness and diameters for both aorta and PA. The results for the different thicknesses (2–2.5 mm) showed that the circumferential stress peaks were found in the range 118–158 kPa for aorta roots and 206–277 kPa for native PA, in line with those obtained by Mookhoek et al. [42,43] in their simulations. A similar comparison can be made in terms of deformed thicknesses and diameters, where the major discrepancies were registered with respect to PA.

This difference can be traced in the mechanical properties of the vessels, in the present case related to native PA (PA implanted before degeneration to expansive systemic load effect), while Mookhoek et al. [42,43] referred to PA autograft already degenerate. Based on the simulations, it is thus expected that substituting native aorta with PA root induces an instantaneous elastic increase in the vessel diameter of about 32–33%, which is associated with a corresponding decrease in thickness of about 23–24%. From a biomechanical point of view, the rapid absorption of polydioxanone constituting the internal part of the device may limit the potential negative effect of excessive stretching and improvement in the steeper curve in the circumferential response. Improvement in the longitudinal stretching of the pulmonary autograft by an external component of the device is indicative of the auxetic effect of expanded polytetrafluoroethylene (e-PTFE). Successful reinforcement with a semi-resorbable device can also be favorable to pulmonary autograft function in patients needing to match somatic growth. The attendant decrease in PA expansion and the preserved features of the valve leaflets enhances the durability of Ross operation.

### 4.2. Clinical Application

Based on the results of this study, we could draw the following conclusions and trace new avenues of future investigation in the field of Ross procedure. First, it is important to reconsider the assumption of a linear relation in the dimensional expansion of the PA structures and how it relates to the age of the PA implant, giving attention to the phase of the somatic growth of the patient. In this context, our findings imply a further analysis of the observations previously described by Horer et al. regarding the yearly diameter increase in the annulus, Valsalva sinus, and sinotubular junction of the PA [14,38,48]. Second, the length of the conduit to be implanted and the technique of implantation need to be specifically tailored.

In fact, when translating these considerations into the clinical practice, the age of implant in the period of somatic growth (before and after the six years) plays a crucial role in this context. Importantly, before six years of age, there is no difference in size between the annulus, Valsalva sinus, and sinotubular junction, and the pulmonary autograft can be considered as a cylinder with homogeneous size, and the miniroot technique appears to be the most appropriate in this case. After six years of age, three different dimensions, annulus, Valsalva sinuses, and sinotubular junction, are defined with supposedly different mechanical resistance to expansion [34,35,38]. Apart from the annulus, which revealed as the less deformable structure in the root, in this study, we observed a consensual increase in longitudinal and circumferential stress and deformation of the PA root using a load of a considerable entity. This indicates a stress-shielding characteristic of the PA root, which allows for a uniform distribution of forces to be imparted within the walls and could guarantee relatively long durability [34,35,38].

Although the study had insufficient power to draw definitive conclusions about the relative effects of the two types of stresses on the pulmonary conduit, we can speculate that the duration of stress application and the rates of dilation might be associated with the length of the conduit implanted. In our experimental setting, we found no significant changes for PA longer than 3.5 cm, but in the actual surgical practice the correct height of the pulmonary autograft to be implanted was difficult to determine in children, and the distal anastomosis was normally performed beyond the level of the neo-sinotubular junction in consideration of the somatic growth. For these reasons, a miniroot technique with reinforcement represents the most appropriate option for this age range, and the use of reinforcing materials that do not impair the compliance of the native PA is important at this stage [14,15,38]. In young adults (>16 years), when the somatic growth phase is completed, a large series demonstrated the importance of reinforcing the PA, showing a 6-fold increase in the reoperation rate in subjects who did not receive PA reinforcement and a linearized occurrence rate (LOR) of reoperation for aortic valve dysfunction of more than 1.8%/patient-year in case of the root replacement technique without reinforcement. In parallel, the use of reinforcement in the miniroot approach could reduce the incidence of aortic regurgitation with LOR 0.32%/patient-year [16,17,18,19]. Other reports, while stressing the significance of measures to stabilize the annulus, demonstrated the superiority of the subcoronary technique in the adult population in terms of autograft durability with freedom from autograft reintervention of 97% at 10 years and 91% at 20 years [5]. We experimentally found a higher degree of resistance to the deformation of the annulus, and the valve leaflet of the PA and reports of the valve-sparing redo operation for PA failure support this observation. This should theoretically encourage the implant of PA in a sub-coronary position and reinforce the concept that pulmonary leaflets do not deteriorate when over-pressurized, although the exact time-to-failure cannot be established with certainty. However, besides the progressive clinical abandon of this technique, our results also revealed its biomechanical limitations as the absence of the entire root determines a non-homogeneous distribution of forces concentrating on few points on the leaflets and resulting in cusp degeneration. This idea is confirmed by the report on the long-term outcomes of the German–Dutch Ross registry, which showed that the leading cause of autograft valve failure with the need for reoperation in the subcoronary group was structural valve deterioration (80% of all reoperations), due to cusp prolapse (69% of all structural valve deteriorations) [5].

Conversely, we can strongly support the advantage of the miniroot technique that preserves geometry and function of the pulmonary valve because it imparts homogenous distribution of circumferential and longitudinal forces within the entire system of root, annulus, and valve.

## 5. Conclusions

The results of this study advance our understanding of the relative benefits of pulmonary autograft for the management of severe aortic valve disease, as demonstrated by Donald Ross 40 years [10,49,50,51]. However, it launches a warning about the importance of the choice of the length of the conduits as mechanical deformation, and, therefore, potential failure, increases with the length of the segment subjected to stress. Strengthening of the distal pulmonary root anastomosis using external reinforcement, modifying the ascending phase of the circumferential stress curve, might be advisable as previously described [32,33,34]. We believe that the data presented herein could provide a basis for the complete understanding of our studies on PA reinforcement and could comprehensively elucidate the clinical benefit and the future expectations of this practice during the Ross operation.

PA is an ideal substitute for aortic valve replacement not only in Mr. Ross’s dreams [49] but also from the bioinspired point of view [4,31,38].
